# How Honey Bee Colonies Survive in the Wild: Testing the Importance of Small Nests and Frequent Swarming

**DOI:** 10.1371/journal.pone.0150362

**Published:** 2016-03-11

**Authors:** J. Carter Loftus, Michael L. Smith, Thomas D. Seeley

**Affiliations:** Department of Neurobiology and Behavior, Cornell University, Ithaca, New York, United States of America; Salford University, UNITED KINGDOM

## Abstract

The ectoparasitic mite, *Varroa destructor*, and the viruses that it transmits, kill the colonies of European honey bees (*Apis mellifera*) kept by beekeepers unless the bees are treated with miticides. Nevertheless, there exist populations of wild colonies of European honey bees that are persisting without being treated with miticides. We hypothesized that the persistence of these wild colonies is due in part to their habits of nesting in small cavities and swarming frequently. We tested this hypothesis by establishing two groups of colonies living either in small hives (42 L) without swarm-control treatments or in large hives (up to 168 L) with swarm-control treatments. We followed the colonies for two years and compared the two groups with respect to swarming frequency, *Varroa* infesttion rate, disease incidence, and colony survival. Colonies in small hives swarmed more often, had lower *Varroa* infestation rates, had less disease, and had higher survival compared to colonies in large hives. These results indicate that the smaller nest cavities and more frequent swarming of wild colonies contribute to their persistence without mite treatments.

## Introduction

In recent decades, beekeepers worldwide have faced numerous challenges in maintaining healthy honey bee (*Apis mellifera*) colonies [1]. Annual mortality rates of over 20% of colonies are now considered typical, with some beekeepers reporting annual losses of up to 90% [2]. A variety of factors have contributed to the elevated rate of colony mortality, but perhaps the most significant is the introduction from Asia of the ectoparasitic mite, *Varroa destructor*, which acts as an efficient vector of the viruses of honey bees [3–5]. The rate of colony loss in Europe and North America nearly tripled after the arrival of *Varroa* in the 1970s and 1980s [6]. These mites have introduced a new viral transmission route that has altered the viral landscape and caused a massive loss of diversity in Deformed Wing Virus (DWV) [7], the pathogen that is linked with the demise of honey bee colonies [8]. Without treatments for *Varroa*, managed honey bee colonies almost always die within two or three years [9,10].

Even though *Varroa* infestations lead to the deaths of honey bee colonies managed by beekeepers unless they are given mite-control treatments, several investigators have reported populations of European honey bee colonies living in the wild that have persisted without mite-control treatments, despite being infested with *Varroa* (Brazil [11], Russia [12], Sweden [10], France [13], and United States [14]). In all of these populations, selective pressures by the mites and viruses have probably produced genetic changes in the bees that give them intrinsic resistance to these parasites and pathogens. We know, for example, that the population of wild colonies in the Arnot Forest in the U.S. experienced massive colony mortality between 1977 and 2010, and that hundreds of the nuclear genes in this population show strong signs of selection [15]. However, there may also be environmental factors that are making it possible for wild colonies to survive mite infestations without mite treatments, when managed colonies cannot. We hypothesized that the relatively small nest cavities of wild colonies might partially explain their greater ability to survive *Varroa* infestations without treatments. In North America, wild honey bees occupy tree cavities with volumes of 30 to 60 L [16], whereas managed colonies are usually housed in hives with volumes of 120 to 160 L so that they have sufficient room to create large honey stores for beekeepers to harvest. Because wild colonies live in small nest cavities, which are conducive to swarming [17], and because they are not subject to beekeeping practices for swarm control, wild colonies probably swarm more often than managed colonies. We also hypothesized that more frequent swarming by wild honey bee colonies, together with their reduced brood rearing (because they have smaller nests), hinders *Varroa* reproduction and so makes these wild colonies less vulnerable to the mites and to the diseases they spread. Not only does casting a swarm export about 35% of a colony’s *Varroa*—because about 70% of the adult bees leave when a colony casts a swarm [18] and approximately 50% of the *Varroa* in a colony are on the adult bees [19]—it also creates a broodless period in the swarming colony. *Varroa* depends on honey bee brood for reproduction, so this broodless period may help further shrink the *Varroa* population in a colony that has swarmed.

To test the hypothesis that small nest cavities contribute to the ability of wild colonies to persist without *Varroa* treatments, we performed an experiment that compared two groups of colonies. In one group, the colonies lived in small (42 L) hives and were left alone. These were our "small-hive colonies," which simulated wild colonies of honey bees. In the other group, the colonies lived in large hives (up to 168 L) and were managed in ways that reduced their swarming and maximized their honey production: queen cells were removed periodically and colonies were given two deep hive bodies for a brood chamber plus another two deep hive bodies ("honey supers") for honey storage. These were our "large-hive colonies", which simulated typical managed colonies of honey bees. We monitored the brood and adult bee populations, mite infestation rates, incidences of disease, occurrences of swarming, honey production, and survival of the colonies in both groups over a two-year period (May 2012–April 2014). We predicted that the small-hive colonies would experience more frequent swarming, lower *Varroa* infestation rates, lower incidences of disease, lower honey production, and higher colony survival than the large-hive colonies.

## Materials and Methods

### Study site

This study was performed at a site owned by Cornell University outside of Ithaca, NY (42°26'9.88"N, 76°25'50.45"W). The site consisted of a field with two mowed areas for two apiaries: one for the small-hive colonies and one for the large-hive colonies. The two apiaries were spaced 60 m apart, center to center. Each apiary had open land to the east, south, and west, and thus received good sun exposure. And each had a windbreak to the north, either a storage building or a grove of spruce trees, and thus was well sheltered. Also, each apiary contained six hive stands for pairs of hives, with each pair separated from its neighboring pair by 4 m.

### Colony establishment and maintenance

On 22 May 2012, we installed in both apiaries 12 nucleus colonies in 5-frame hives. Each nucleus colony's hive contained 5 full-depth Langstroth frames (48 x 23 cm): 2 frames of comb—one filled with brood, one partially filled with pollen and honey—covered with adult bees, 1 frame of comb filled with honey but without bees, 1 frame of empty comb, and 1 frame of beeswax comb foundation. We obtained the frames of bees and brood for the 24 nucleus colonies from 12 source colonies living in an apiary 4.4 km away. We took 4 frames of bees and brood from each source colony, so each source colony provided the bees and brood for one colony in both the small-hive and the large-hive treatment groups. This ensured that the two treatment groups started out with the same average *Varroa* infestation rate of adult bees. Every frame of comb used in this study had minimal drone comb (< 10 cm^2^). Each nucleus colony was given an open-mated Italian queen bee purchased from Olivarez Honey Bees, Inc (Chico, California) and all 24 queens were accepted. All were then marked with a dot of yellow paint on the thorax.

On 5 June 2012, we transferred all the colonies in both apiaries from their 5-frame hives into 10-frame Langstroth hives, each of which had a volume of 42 L. Adjacent colonies were given different colored hives, to minimize drifting of workers and drones between colonies. To fill each colony’s 10-frame hive with combs, we gave it 4 more frames of empty comb and one more frame of comb containing honey. We also installed an entrance reducer in each hive so each one had a small, 15-cm^2^-entrance opening.

On 5 July 2012, we inspected the colonies and gave additional bees or brood, or both, to the three smallest colonies in each group, to bring all the colonies up to the same strength. Specifically, we gave one colony in each treatment group 2 frames that were filled with capped brood but were not covered with worker bees, and we gave 2 colonies in each group 2 frames that were both filled with capped brood and covered with worker bees. Also, because we found that one colony in each treatment groups had a poor queen (i.e., one that was laying few eggs), we removed the failing colony in each treatment group and replaced it with a thriving colony that was size-matched to the other colonies in the study. These two replacement colonies, and the frames of bees and brood that we used to equalize the colonies, all came from the same apiary that provided the bees for establishing the study colonies. A few days later, on 9 July 2012, we placed a second 10-frame hive body containing 6 frames of empty, drawn comb and 4 frames of beeswax comb foundation on top of each hive in only the large-hive treatment group. In late July there was a prolonged nectar dearth, so on 23 July 2012 we fed each colony 2.5 L of 50:50 (vol/vol) sugar syrup using a division board feeder.

On 20 September 2012, we installed in each hive's entrance a screen (1.27 cm wire mesh) that prevented mice from entering over winter, and we gave 5 of the large-hive colonies that had not stored enough honey to survive winter 1–4 frames of comb filled with capped honey.

On 4 May 2013, we found that three of the small-hive colonies had died over the winter, so we replaced them. Each of the three replacement colonies had 6 frames of comb covered with bees (of which 4–5 contained brood), thus these three colonies were size-matched to the surviving colonies in the small-hive treatment group. The three replacement colonies came from an apiary of colonies that had not been treated for mites the previous summer, so they were matched to the surviving colonies in the small-hive treatment group in this regard too. We found that all of the large-hive colonies had survived winter, but that one was markedly weaker than the others (with bees and brood on just 3 frames of comb), so we gave this colony 3 frames of comb that were covered with bees and contained capped brood, to bring it up to the strength of the other large-hive colonies. On 5 May 2013, we removed from all the hives the screens that prevented mice from entering the hives, and we gave all the large-hive colonies a third deep hive body containing 10 frames of drawn comb.

On 27 May 2013, we gave all the large-hive colonies a fourth deep hive body containing 10 frames of plastic foundation that we had coated thickly with melted beeswax. At this point, each colony in the large-hive treatment group occupied a 168-L hive. The colonies in the small-hive treatment group continued to occupy their original 42-L hives (a single hive body), as they would for the duration of the study.

To further differentiate the colonies living in the small hives, which mimicked the nests of colonies living in the wild, from the colonies living in the large hives, which were typical for colonies managed for honey production, we manipulated the large-hive colonies in ways that reduced their likelihood of swarming and boosted their honey production. These manipulations consisted of adding hive bodies (to provide "honey supers") as already described, dispersing frames of brood among hive bodies (to reduce brood nest congestion) when we provided the fourth hive body on 27 May 2013, and destroying all queen cells (to inhibit swarming) found during the colony inspections on 4 May 2013 and 5 June 2013. It should be noted, however, that because the colonies in the study were inspected only once a month, they all still had plenty of opportunities to rear queens and swarm.

On 28 August 2013, we harvested honey from the large-hive colonies. To do so, we removed the top-most hive body (honey super) from each of the 12 hives, extracted the honey, and then returned each hive body, with its frames now largely empty of honey, to its colony. We weighed the 12 hive bodies before and after extracting the honey and determined that we had harvested a total of 219 kg of honey. No more honey was removed from any of the study colonies, to help ensure that they would have large stores of honey for winter.

On 9 October 2013, we again installed in each hive's entrance a screen (1.27 cm wire mesh) that prevented mice from entering over winter.

Table 1 summarizes the colony treatments made during the two years of the study.

**Table 1:** Summary of treatments of the 24 colonies over the course of the study.

Date	Treatment
22 May 2012	Established 24 nucleus colonies in 5-frame hives.
5 June 2012	Transferred all colonies to 10-frame hives with deep frames.
5 July 2012	Gave additional bees and brood to 3 colonies, in both groups. Also, replaced 1 colony with a failing queen with a size-matched colony with a thriving queen, in both groups.
9 July 2012	Gave each LH colony a 2nd deep hive body containing 10 frames with drawn comb.
23 July 2012	Fed each colony 2.5 L of 50:50 (vol:vol) sugar syrup.
20 Sept 2012	Gave 1–4 frames of honey to 5 of the LH colonies. Installed mouse screens.
4 May 2013	Examined all colonies. Cut out swarm/queen cells in the LH colonies. Gave all LH colonies a 3rd deep hive body (1st honey super) containing frames of drawn comb
5 May 2013	Replaced 3 colonies in the SH group that died over winter. Strengthened 1 colony in the LH group with 3 frames of bees and brood
27 May 2013	Gave LH colonies a 4th deep hive body (2nd honey super) containing 10 frames with plastic foundation heavily coated with beeswax (more storage space needed because of strong honey flow from black locust, *Robinia pseudoacacia*.) 4 frames of brood in the 2nd hive body swapped with 4 frames of empty comb in the third hive body, to reduce broodnest congestion.
5 June 2013	Examined all colonies, cutting out swarm/queen cells in the LH colonies.
28 Aug 2013	Removed 1 honey super from each of the 12 LH colonies and extracted the honey (219 kg).
9 Oct 2013	Installed mouse screens.

SH colony, small-hive colony; LH colony, large-hive colony.

### Measurements

Using standard methods [20], we made systematic measurements of each colony's adult bee population, number of cells of brood, and mite infestation rate of the adult bees. To measure the adult bee population, we examined each side of every frame in a hive and estimated to the nearest 10% the fraction of the frame covered by adult bees. These values were summed for a colony and then multiplied by 1,000 to obtain an estimate of the adult bee population of the colony; one side of a frame that is fully, but not densely, covered by bees has approximately 1,000 bees [20]. Data on the number of cells of brood in a colony were obtained similarly, estimating to the nearest 10% the fraction of the cells on each side of every frame that were filled with brood (eggs, larvae, and pupae). These values were summed and multiplied by 3,276 to give the total number of cells containing brood in a hive, because one side of a frame contains approximately 3,276 cells [20]. In the first year, 2012, we took measurements of colony size on 5 June and 5 July to check that the small-hive and large-hive colonies started out with the same mean numbers of adult bees and cells of brood, which they did (see Results). In the second year, 2013, we took these measurements every month from May to September, in the middle of each month.

We made measurements of the mite infestation rate of the adult bees (mites per 100 bees) in each colony using the powdered sugar method [21]. On 19 July 2012, we measured the mite infestation rate of each colony in both treatment groups to check that the colonies in the two groups began the study with the same mean infestation rates, which they did (see Results). In 2013, we measured the mite infestation rate in each colony in the middle of each month from May to October.

While taking the monthly measurements of colony size and mite infestation rate, we made visual inspections for diseases (deformed wing virus, chalkbrood, sacbrood, and American foul brood) present in each colony, using the criteria described by Hansen [22]. We also noted if a colony had swarmed, which was indicated by various signs: a break in the colony’s brood production, an unmarked queen, or the presence of a queen cell from which a queen had recently emerged. (Note: the presence of an unmarked queen in a colony can through queen supersedure as well as swarming, but we believe that most of the unmarked queens found in this study were due to swarming because we collected numerous swarms (13 total) from the trees and shrubs near the two apiaries.) In the third year, on 20 April 2014, we made a final inspection of the colonies to see which had survived their second winter.

### Statistical tests

The average values for adult bee population, number of cells of brood, and mites per 100 adult bees were calculated for both the small-hive and large-hive colonies after every bout of data collection. The averages were compared for statistical significance using a one-tailed Student’s t Test, with a Bonferroni correction applied (alpha = 0.01 rather than 0.05) because we made repeated tests of the differences between treatment groups in adult bee populations, brood cell counts, and mite density measurements. All descriptive statistics are given as the mean ± 1 SE.

One-tailed Fisher's Exact Probability Tests were used for tests of association between hive size and the occurrence of swarming, presence of bees with shriveled wings caused by the deformed wing virus, and incidence of colony mortality.

## Results

### Colony size and swarming

**Fig 1:**
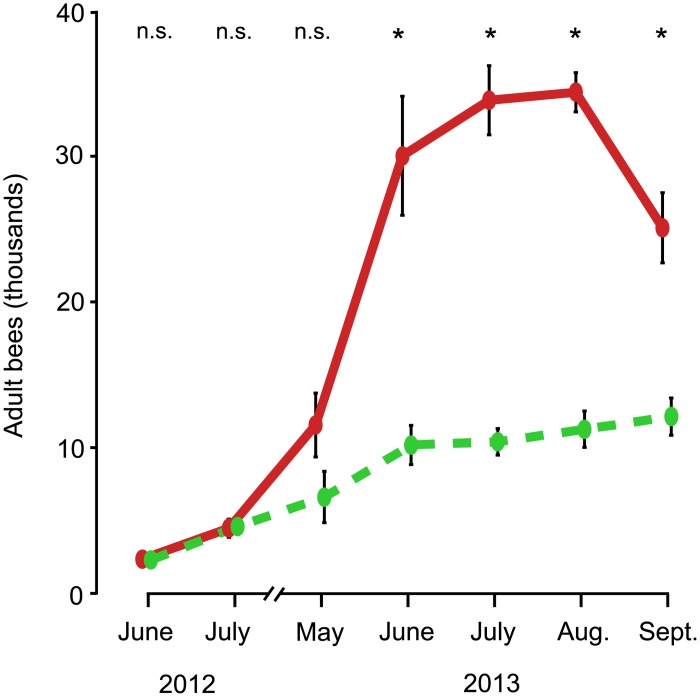
Dynamics of the adult bee population in colonies housed in small hives (dashed, green line) and colonies housed in large hives (solid, red line), from June 2012 to September 2013. Error bars represent ± 1 SE. Asterisks denote significant differences between values (p < 0.01).

**Fig 2:**
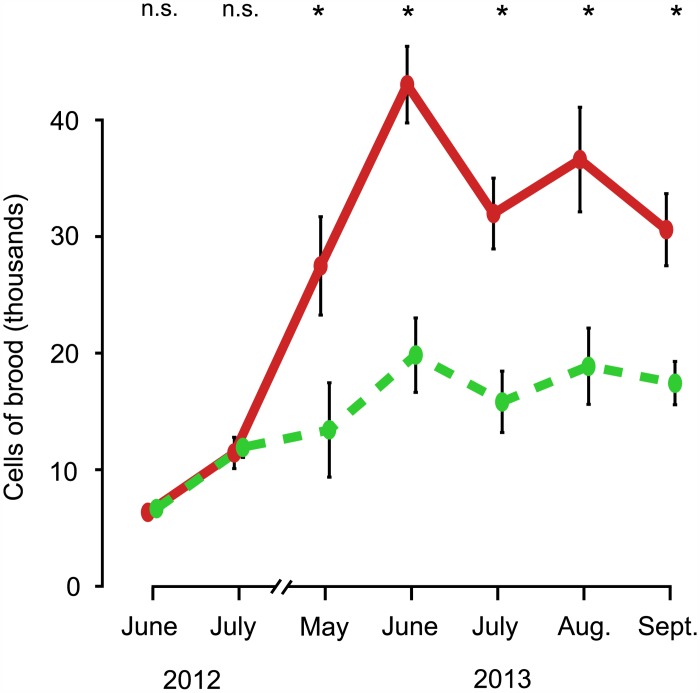
Dynamics of the amount of brood in colonies housed in small hives (dashed, green line) and colonies housed in large hives (solid, red line), from June 2012 to September 2013. Error bars represent ± 1 SE. Asterisks denote significant differences between values (p < 0.01).

As shown in Figs 1 and 2, the colonies in the two treatment groups started out in June 2012 with essentially the same mean number of adult bees (small-hive: 2,417 ± 219, large-hive: 2,479 ± 193, p = 0.39) and nearly the same mean number of cells of brood (small-hive: 6,579 ± 480, large-hive: 6,279 ± 407, p = 0.34). This absence of a difference between the two groups continued into July 2012 for both the adult bee population (small-hive: 4,725 ± 414, large-hive: 4,604 ± 625, p = 0.44) and the brood cell count (small-hive: 11,689 ± 872, large-hive: 11,381 ± 1,344, p = 0.39).

By May 2013, however, the large-hive colonies, compared to the small-hive colonies, had substantially (but not significantly) more adult bees (small-hive: 6,744 ± 1,765, large-hive: 11,700 ± 2,198, p < 0.10) and significantly more cells of brood (small-hive: 13,359 ± 4,058, large-hive: 27,464 ± 4,230, p < 0.01). Both the adult bee population and the brood cell count were significantly higher in the large-hive colonies than in the small-hive colonies for the remainder of the study. BEES: June, small-hive: 10,325 ± 1,346, large-hive: 30,208 ± 4,091, p < 0.001; July, small-hive: 10,542 ± 913, large-hive: 34,025 ± 2,376, p < 0.0001; August, small-hive: 11,408 ± 1,251, large-hive: 34,575 ± 1,349, p < 0.0001; September, small-hive: 12,275 ± 1,278, large-hive: 25,258 ± 2,413, p < 0.0001. BROOD CELLS: June, small-hive: 19,793 ± 3,200, large-hive: 43,052 ± 3,294, p < 0.001; July, small-hive: 15,779 ± 2,638, large-hive: 31,968 ± 3,045, p < 0.001; August, small-hive: 18,837 ± 3,280, large-hive: 36,609 ± 4,497, p < 0.01; September, small-hive: 17,390 ± 1,864, large-hive: 30,576 ± 3,099, p < 0.001.

None of the colonies in either treatment group swarmed (i.e., had a change of queen) in 2012. In 2013, however, fully 10 of the 12 small-hive colonies swarmed, but only 2 of the 12 large-hive colonies swarmed. Thus, the small-hive colonies exhibited a significantly higher frequency of swarming than the large-hive colonies: one-tailed Fisher's Exact Probability Test, p < 0.002.

### *Varroa* infestation rates on adult bees

**Fig 3:**
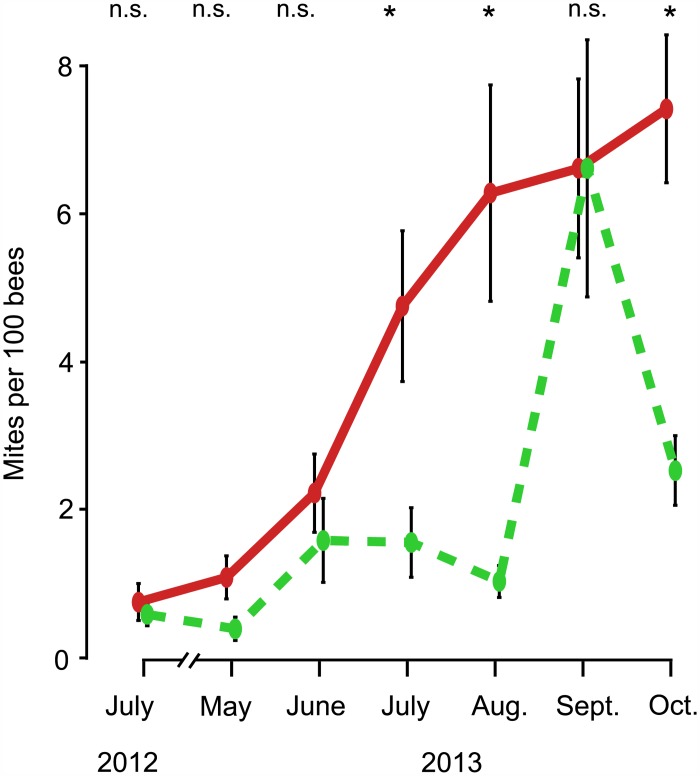
Dynamics of *Varroa* infestation rates on adult bees in colonies housed in small hives (dashed, green line) and colonies housed in large hives (solid, red line), from July 2012 to October 2013. Error bars represent ± 1 SE. Asterisks denote significant differences between values (p < 0.01).

As shown in Fig 3, the colonies in the two treatment groups had approximately the same average mite count per 100 bees in 2012 (July, small-hive: 0.58 ± 0.15, large-hive: 0.75 ± 0.25, p = 0.29). In May and June 2013, however, the average mite counts per 100 bees began to grow higher in the large-hive colonies relative to the small-hive colonies (May, small-hive: 0.39 ± 0.16, large-hive: 1.08 ± 0.29, p < 0.03; June, small-hive: 1.58 ± 0.58, large-hive: 2.22 ± 0.53, p <0.20). At this point, the mite infestation rates rose rapidly in the large-hive colonies, so that their mean values were significantly higher than those of the small-hive colonies from July to October, except in September, when the small-hive colonies experienced a large, transient spike in their mean mite infestation rate (July, small-hive: 1.56 ± 0.47, large-hive: 4.75 ± 1.02, p < 0.01; August, small-hive: 1.03 ± 0.22, large-hive: 6.28 ± 1.46, p < 0.01; September, small-hive: 6.61 ± 1.74, large-hive: 6.61 ± 1.21, p = 0.49; October, small-hive: 2.53 ± 1.63, large-hive: 7.42 ± 1.00, p <0.001).

### Colony disease and mortality

The first sign of disease in the 24 study colonies came in mid-August 2013 when a bee with severely deformed wings was spotted in a large-hive colony. By-mid September 2013, this colony had lost its queen and had collapsed; its hive was nearly empty of bees. At this time, we also found three other large-hive colonies that contained bees with shriveled wings, symptomatic of high levels of the deformed wing virus (DWV). Two of these three colonies also showed signs of sacbrood virus. By mid October, three more large-hive colonies contained bees with shriveled wings, so 7 out of the 12 colonies in this group showed symptoms of high levels of DWV. We observed no symptoms of DWV in the small-hive colonies throughout this study, so the large-hive colonies exhibited a significantly higher incidence of this disease (7 out of 12) than the small-hive colonies (0 out of 12): one-tailed Fisher's Exact Probability Test, p < 0.002.

Regarding mortality, as mentioned already, one large-hive colony collapsed and died in September 2013. One small-hive colony also died in September 2013 but not from disease; its queen began to lay only unfertilized (drone producing) eggs. Nine more large-hive colonies died between October 2013 and April 2014, as did 3 more small-hive colonies. Thus the large-hive colonies experienced significantly higher mortality (10 out of 12) than the small-hive colonies (4 out of 12): one-tailed Fisher's Exact Probability Test, p < 0.037.

## Discussion

The results of this study support the hypothesis that the persistence of wild colonies is aided by their habits of nesting in small cavities and swarming frequently. By the end of the second summer of the study, the colonies living in small hives had a mean *Varroa* infestation rate of adult bees that was only about one third of that found in the colonies living in large hives (Fig 3). Moreover, while none of the small-hive colonies showed signs of disease, seven of the 12 large-hive colonies showed symptoms of high infection with the deformed wing virus (DWV), which is closely associated with a high infestation of *Varroa* [23]. Furthermore, all seven of the colonies with symptoms of high infections of DWV died by April 2014. It seems clear that the colonies living in the large hives were more susceptible to *Varroa* than the colonies living in the small hives; the higher *Varroa* infestation rates impaired their health and survival, so that in the end only 2 out of 12 large-hive colonies were still alive. In contrast, the small-hive colonies had relatively low *Varroa* infestation rates, did not show symptoms of high DWV infections, and had better survival with 8 out of 12 colonies still alive at the end of the study.

One curious finding in this study was the transient spike in the mean *Varroa* infestation rate in colonies in the small-hive group in mid-September 2013. This spike occurred because 3 of the 12 small-hive colonies suddenly showed surprisingly high mite infestation rates: 15–17 mites/100 bees. These 3 colonies had only 1–3 mites/100 bees in mid-August. It is probably not just a coincidence that shortly before this spike was recorded, one of the large-hive colonies had developed a high *Varroa* infestation rate (13 mites/100 bees) and had collapsed. There were piles of dead bees in front of this colony's hive and the hive was nearly empty of bees, except for bees robbing honey. Presumably the robbing bees came from nearby colonies. Robbing of a dying or dead colony's honey is not unusual [24], especially if the colony’s decline coincides with a nectar dearth [25], which was the situation when the colony in the large-hive treatment group collapsed in late August and early September. Because *Varroa* can climb onto robber bees [25,26,27], and indeed are increasingly apt to climb onto the foragers/robbers (not just the nurse bees) in a colony as the *Varroa* abundance increases [28], it is likely that the spike in the mean *Varroa* infestation rate in the small-hive colonies in mid-September was due to importation of mites by small-hive colonies that had robbed honey from the collapsing large-hive colony. If so, then this spike in the *Varroa* infestation rate in the small-hive colonies, which were living only 60 m from the large-hive colonies, is an artifact of the experimental setup.

This spike in *Varroa* infestation rate may explain why there was considerable colony mortality (4 out of 12 colonies) in the small-hive group. One small-hive colony died because its queen ran out of sperm, so eventually only drones were produced in this colony. Interestingly, the other 3 small-hive colonies that died were the 3 colonies that experienced strong spikes in their *Varroa* infestation rates during September. Thus the possible importation of *Varroa* (and associated viruses) from the collapsing large-hive colony in September may have led to the deaths of these three small-hive colonies over the winter. Because robber bees probably transfer diseases mainly between hives in close proximity [25], it is regrettable that we did not space the two apiaries for the two treatment groups farther apart. A recent study [29] of the effects of colony crowding has shown that crowding renders colonies more likely to acquire high infestations of *Varroa* in late summer, when robbing behavior is most common.

We performed this study to investigate how populations of wild colonies are able to survive without *Varroa* treatments whereas managed colonies rarely persist for more than two to three years without being treated for *Varroa*. Our results suggest that the small size of the nesting cavities of wild colonies is helping them persist, despite having infestations of *Varroa*. As we predicted, the colonies in this study that lived in small hives and were not given swarm-prevention treatments tended to swarm, but the colonies that lived in large hives and were given swarm-prevention treatments tended to not swarm. This greater swarming in the small-hive colonies, which evidently arose from the more crowded conditions in their smaller hives [17,30], meant that more of the small-hive colonies experienced mid-summer breaks in their brood rearing. Because a swarming event exports about 35% of a colony’s *Varroa* [18,19] and temporarily deprives *Varroa* of the pupal brood it needs for its reproduction [31], it seems likely that having a higher rate of swarming helps wild colonies limit their *Varroa* infestations and thereby survive and reproduce well enough to maintain a population of wild colonies in a region. It is also possible that nesting in a small cavity helps the bees avoid high *Varroa* infestation rates because colonies with small nests possess fewer cells of brood and thereby provide *Varroa* with fewer opportunities for reproduction (Fig 2).

The results of this study point to a management practice whereby colonies could be housed in large hives, manipulated for honey production, not be treated with miticides, and yet not succumb to *Varroa* and associated viruses. Specifically, this study suggests that splitting colonies—a practice in which the queen and a portion of the adult bees and brood are removed from a colony and placed in another hive to produce an additional colony, meanwhile the original colony rears a replacement queen—might be an effective way to reduce mite populations in large colonies managed for honey production [32]. The splitting of a colony results in a broodless period in the colony, and this may limit the infestation rate of *Varroa* in managed colonies in the same way that swarming evidently does in wild colonies. It may also, however, depress a colony's honey production. We suggest that further research should be done on the use of colony splitting as a non-chemical method for reducing *Varroa* in managed honey bee colonies.
